# Anaplasmataceae circulation in wild mammals and ticks from Goiás state, midwestern Brazil

**DOI:** 10.1590/S1984-29612026027

**Published:** 2026-07-20

**Authors:** Raphaela Bueno Mendes Bittencourt, Ana Cláudia Calchi, Luiz Gustavo Schneider de Oliveira, Fabiana Marques Boabaid, Lucianne Cardoso Neves, Nicolas Jalowitzki de Lima, Ennya Rafaella Neves Cardoso, Gabriel Cândido dos Santos, Lucas Lisboa Nunes Bonifácio, Filipe Dantas-Torres, Leo Caetano Fernandes da Silva, Marcos Rogério André, Felipe da Silva Krawczak

**Affiliations:** 1 Universidade Federal de Goiás – UFG, Escola de Veterinária e Zootecnia – EVZ, Setor de Medicina Veterinária Preventiva, Laboratório de Doenças Parasitárias – LADOPAR, Goiânia, GO, Brasil; 2 Universidade Estadual Paulista – UNESP, Faculdade de Ciências Agrárias e Veterinárias – FCAV, Departamento de Patologia, Reprodução e Saúde Única, Vector-Borne Bioagents Laboratory – VBBL, Jaboticabal, SP, Brasil; 3 Fundação Oswaldo Cruz – FIOCRUZ, Instituto Aggeu Magalhães, Recife, PE, Brasil; 4 Instituto Brasileiro do Meio Ambiente e dos Recursos Naturais Renováveis – IBAMA, Centro de Triagem de Animais Silvestres de Goiás – CETAS-GO, Goiânia, GO, Brasil

**Keywords:** Anaplasma, capybara, deer, hemoparasites, tick-borne diseases, wildlife, Anaplasma, capivaras, cervídeos, hemoparasitos, doenças transmitidas por carrapatos, animais silvestres

## Abstract

The Anaplasmataceae family comprises vector-borne alpha-proteobacteria with significant impact on animal and human health. In Brazil, the diversity and circulation of these agents in wild mammals require further exploration. This study investigated the presence of Anaplasmataceae agents in wild mammals, and ticks from Goiás state, in midwestern Brazil. A total of 97 blood samples, 22 tissue samples, and 69 ticks from 18 mammalian species were analyzed. Conventional PCR targeting the 16S rRNA gene revealed Anaplasmataceae DNA in 11.3% (11/97) of blood samples, 13.6% (3/22) in tissue samples and 2.9% (2/69) of tick specimens. Positive hosts included *Subulo gouazoubira*, *Hydrochoerus hydrochaeris*, *Cerdocyon thous*, *Chrysocyon brachyurus*, *Tapirus terrestris*, and *Myrmecophaga tridactyla*, as well as two *Amblyomma sculptum* ticks. Phylogenetic analysis based on the 16S rRNA gene from *Anaplasma* sp. detected in *S. gouazoubira* clustered with “*Candidatus* Anaplasma boleense”, while 23S-5S rRNA (intergenic spacer) ITS supported a potentially undescribed Anaplasmataceae agent. A short 16S rRNA gene fragment showing high identity with “*Ca*. Anaplasma boleense” was detected in *H. hydrochaeris* for the first time. These findings expand current knowledge on the diversity and host range of Anaplasmataceae in Brazil, highlighting the role of wild mammals in maintaining these bacteria in Goiás state, midwestern Brazil.

## Resumo

A família Anaplasmataceae compreende alfa-proteobactérias com impacto significativo na saúde animal e humana. No Brasil, a diversidade e a circulação desses agentes em mamíferos silvestres ainda necessitam de maior investigação. Este estudo investigou a presença de agentes Anaplasmataceae em mamíferos silvestres e carrapatos do estado de Goiás, no Centro-oeste do Brasil. Um total de 97 amostras de sangue, 22 amostras de tecidos e 69 carrapatos provenientes de 18 espécies de mamíferos foram analisadas. A PCR convencional direcionada ao gene 16S rRNA revelou DNA de Anaplasmataceae em 11,3% (11/97) das amostras de sangue, 13,6% (3/22) das amostras de tecidos e 2,9% (2/69) dos carrapatos. Os hospedeiros positivos incluíram *Subulo gouazoubira*, *Hydrochoerus hydrochaeris*, *Cerdocyon thous*, *Chrysocyon brachyurus*, *Tapirus terrestris* e *Myrmecophaga tridactyla*, além de dois carrapatos *Amblyomma sculptum*. A análise filogenética do gene 16S rRNA de *Anaplasma* sp. detectado em *S. gouazoubira* agrupou-se com “*Candidatus* Anaplasma boleense”, enquanto o sequenciamento da região ITS 23S-5S rRNA sustentou um potencial agente não descrito de Anaplasmataceae. Além disso, um fragmento curto do gene 16S rRNA com alta identidade com “*Ca.* Anaplasma boleense” foi detectado em *H. hydrochaeris* pela primeira vez. Os achados ampliam o conhecimento sobre Anaplasmataceae em mamíferos silvestres no Centro-oeste brasileiro, esclarecendo o papel de mamíferos silvestres na manutenção dessas bactérias no estado de Goiás, centro-oeste brasileiro.

## Introduction

Tick-borne diseases are among the most significant infectious diseases of public health concern, with an increasing incidence worldwide (Dantas-Torres et al., 2012). In this context, bacteria from the Anaplasmataceae family are notable for their significant impact on animal and human health (André, 2018). These agents are responsible for diseases such as bovine anaplasmosis and canine monocytic ehrlichiosis, and are well known for their zoonotic potential (Dumler, 2005; Stuen et al., 2013). This bacterial family includes the genera *Ehrlichia*, *Anaplasma*, *Wolbachia*, *Neorickettsia*, *Neoehrlichia*, *Aegyptianella* and “*Candidatus* Allocryptoplasma” (André, 2018; Dumler et al., 2001; Ouass et al., 2023).

Among the Anaplasmataceae species, *Ehrlichia* spp. and *Anaplasma* spp. are particularly relevant to veterinary and human medicine, with significant implications under the One Health implications, as they circulate in complex cycles involving domestic animals, wildlife reservoirs, and tick vectors (Dumler et al., 2001; Nakao, 2025). In veterinary medicine, *Ehrlichia canis* and *Anaplasma platys* are the etiological agents of canine monocytic ehrlichiosis (CME) and canine cyclic thrombocytopenia worldwide (De Tommasi et al., 2014; Moraes-Filho et al., 2015). Furthermore, zoonotic agents such *Anaplasma phagocytophilum* and *Ehrlichia chaffeensis* are the primary causative agents of human granulocytic anaplasmosis (HGA) and human monocytic ehrlichiosis (HME) (Cândido et al., 2023; Dumler, 2005; Dumler et al., 1993; Paddock & Childs, 2003; Walker, 2005).

In Brazil, Anaplasmataceae species have been identified in wild mammals (André, 2018; Mongruel et al., 2017; Perles et al., 2022; Soares et al., 2017; Sousa et al., 2017), indicating their circulation in natural ecosystems. Wild animals may act as hosts that contribute to the maintenance of these agents in the environment. Ixodid ticks, the main vectors of these agents, transmit Anaplasmataceae bacteria through blood feeding. Wild mammals serve as important hosts for ticks and often exhibit high infection rates, frequently harboring co-infections with multiple hemoparasites, and they can also serve as sentinels of environmental exposure to infected vectors (Braga et al., 2018; André et al., 2022).

Despite advances in our understanding of tick-borne diseases, significant gaps remain regarding the eco-epidemiology of Anaplasmataceae infections and the role of wild mammals in maintaining and spreading these agents. Addressing these gaps is crucial for implementing effective conservation strategies, better understanding parasite-host interactions, and identifying circulating pathogens, particularly in biodiversity hotspots in Brazil, such as the Cerrado, the predominant biome in the midwestern region of the country (Kassas, 2002; Dantas-Torres et al., 2012).

Therefore, this study aimed to investigate the occurrence of Anaplasmataceae agents in wild mammals and in ticks collected from these hosts at wildlife rescue centers and veterinary clinics in the state of Goiás, midwestern Brazil.

## Material and Methods

### Ethical aspects

The study was approved by the Institutional Animal Care and Use Committee of the Federal University of Goiás (CEUA/UFG; protocol no. 122/22). The collection of biological samples was carried out during routine clinical procedures or handling activities already scheduled within the management protocols of the facilities where the animals were housed. Therefore, specific authorization from the Chico Mendes Institute for Biodiversity Conservation (ICMBio) was not required, as determined by the Biodiversity Authorization and Information System (SISBIO).

### Wild mammals biological samples

Between April 2023 and January 2024, a total of 97 specimens from 18 mammalian species, from different locations in Goiás state, midwestern Brazil, were sampled for blood collection (Table 1). These included wildlife rehabilitation and screening centers of Goiás (CETAS-GYN) and the municipality of Caldas Novas (CETRAS-CN), the Environmental Secretariat of the municipality of Anápolis (SMA), veterinary clinics for unconventional pets, and the Goiânia Zoo (ZOO-GYN). Detailed sampling information, including the number of individuals per species and collection site, is described by Bittencourt et al. (2025). Most of the samples were collected at CETAS-GYN (n=87), followed by CETRAS-CN (n=5), SMA (n=1), veterinary clinics (n=1), and the Goiânia Zoo (n=3).

**Table 1:** Number of blood samples from wild mammals positive for Anaplasmataceae DNA, according to host species, in the state of Goiás, Midwestern Brazil, April 2023 to January 2024.

**Mammal species** ^*^	**No. sampled per species (% of mammals collected)**	**Anaplasmataceae PCR: No. Positive samples/ No. tested (% positive)**
*Alouatta caraya*	11 (11.3)	0/11 (0)
*Callithrix penicillata*	1 (1)	0/1 (0)
*Cerdocyon thous*	11 (11.3)	1/11 (9.1)
*Chrysocyon brachyurus*	6 (6.2)	1/6 (16.7)
*Coendou prehensilis*	6 (6.2)	0/6 (0)
*Didelphys albiventris*	5 (5.1)	0/5 (0)
*Euphractus sexcinctus*	1 (1)	0/1 (0)
*Hydrochoerus hydrochaeris*	4 (4.1)	2/4 (50)
*Leopardus pardalis*	1 (1)	0/1 (0)
*Mazama americana*	1 (1)	0/1 (0)
*Myrmecophaga tridactyla*	21 (21.6)	1/21 (4.8)
*Nasua nasua*	1 (1)	0/1 (0)
*Pecari tajacu*	1 (1)	0/1 (0)
*Puma concolor*	6 (6.2)	0/6 (0)
*Sapajus libidinosus*	4 (4.1)	0/4 (0)
*Subulo gouazoubira*	12 (12.3)	5/12 (41.6)
*Tamandua tetradactyla*	1 (1)	0/1 (0)
*Tapirus terrestris*	4 (4.1)	1/4 (25)
TOTAL	97 (100)	11/97 (11.3)

* Detailed information on animal collection sites is provided in Bittencourt et al. (2025). No: number.

The most represented species were *Myrmecophaga tridactyla* (giant anteater, n=21), *Subulo gouazoubira* (gray brocket deer, n=12), *Alouatta caraya* (black howler monkey, n=11), *Cerdocyon thous* (crab-eating fox, n=11), *Chrysocyon brachyurus* (maned wolf, n=6), *Puma concolor* (puma, n=6), and *Coendou prehensilis* (Brazilian porcupine, n=6), *Didelphis albiventris* (white-eared opossum, n=5). Additional sampled species included *Tapirus terrestris* (lowland tapir, n=4), *Hydrochoerus hydrochaeris* (capybara, n=4), and *Sapajus libidinosus* (bearded capuchin, n=4). Less frequently sampled species were *Callithrix penicillata* (black-tufted marmoset, n=1), *Leopardus pardalis* (ocelot, n=1), *Mazama americana* (red brocket deer, n=1), *Euphractus sexcinctus* (six-banded armadillo, n=1), *Nasua nasua* (coati, n=1), *Pecari tajacu* (collared peccary, n=1) and *Tamandua tetradactyla* (southern tamandua, n=1).

After the procedures for blood collection, the samples were packed using commercial tubes containing ethylenediaminetetraacetic acid (EDTA) for PCR analysis and stored at −20 °C until testing. All samples were transported to the Laboratory of Parasitic Diseases (LADOPAR) at the School of Veterinary and Animal Science, Federal University of Goiás (EVZ/UFG), for laboratory analysis.

Additionally, tissue samples (n=22) were obtained during necropsies of animals from the CETAS-GYN and stored at −20 °C until DNA extraction. These samples were collected from ten animals, including: one *P. concolor* (lung, liver, and spleen), two *C. prehensilis* (spleen), one *T. terrestris* (lung, liver, and spleen), one *Didelphis albiventris* (liver and spleen), two *H. hydrochaeris* (one with lung, liver, and spleen; the other with liver and spleen only), one *C. thous* (lung and liver), and two *C. brachyurus* (one with lung, liver, and spleen; the other with liver and spleen only). Blood samples from all these animals were also included in the analysis.

The animals originated from 30 municipalities in Goiás, as well as two cities located on the border with the state of Mato Grosso do Sul, as described by Bittencourt et al. (2025). However, the exact duration of captivity prior to sampling was not available for all individuals, limiting our ability to determine whether infections were acquired in the wild or during captivity.

### Microscopic analysis of blood smears

Among the 97 blood samples analyzed, 33% (32/97) were fresh and stored at refrigeration temperatures (at 2-8 °C) shortly after collection. Due to the requirement for fresh samples for cytological analysis, duplicate blood smears were prepared and stained, exclusively from these 32 fresh samples, using a commercial rapid stain kit (Panótico Rápido, Laborclin^®^, Pinhais, PR, Brazil). These slides were then subjected to cytological evaluation under a light microscope (Olympus BX41, Olympus Optical Co., Ltd., Tokyo, Japan) at 1000× magnification.

### Tick collection and morphological identification

Ticks were collected from individual hosts, and identified using taxonomic keys (Clifford et al., 1961; Martins et al., 2010; Dantas-Torres et al., 2019), previously conducted as described in Bittencourt et al. (2025). A total of 300 ticks were collected and identified from 37 individual mammals representing nine species: *M. tridactyla* (n = 13), *T. terrestris* (n = 4), *P. concolor* (n = 4), *H. hydrochaeris* (n = 5), *C. prehensilis* (n = 3), *T. tetradactyla* (n = 4), *C. thous* (n = 1), *A. caraya* (n = 1), and *S. gouazoubira* (n = 2).

*Amblyomma sculptum* was the predominant species, accounting for 196 specimens (65.3%) across multiple host species and collection sites, as described in Bittencourt et al. (2025). For molecular analysis, a representative subset of 69 ticks (23% of the total sampled) was selected to encompass the most prevalent tick species, different host taxa, collection sites, and developmental stages. Larval specimens were excluded from molecular screening due to the difficulty of obtaining sufficient DNA for individual analysis. The subset included 59 *A. sculptum* (15 males, seven females, and 37 nymphs), 10 adult ticks from less abundant species, five adult males of *Amblyomma dubitatum*, and five adult males of *Amblyomma nodosum,* were included to account for interspecific diversity and were analyzed individually by molecular techniques. The selection strategy prioritized the most prevalent tick species found on the majority of hosts, in order to include different species, host taxa, collection sites, and developmental stages. All selected ticks were analyzed individually using molecular techniques.

### DNA extraction and PCR for endogenous genes

All blood samples were subjected to DNA extraction from 200 µL of whole blood using a commercial kit (Blood Genomic Prep Mini Spin Kit, Cytiva, Marlow, UK). Additionally, approximately 10 mg of tissue fragments were processed for DNA extraction using the DNeasy Blood and Tissue Kit (Qiagen, Valencia, Santa Clarita, CA, USA). All procedures were performed according to the manufacturers’ instructions, as previously described by Bittencourt et al. (2025). Tick total DNA was extracted using the guanidine isothiocyanate-phenol technique (Sangioni et al., 2005). Negative extraction controls (tubes containing only distilled water) were included in each batch to monitor cross-contamination. Extracted DNA was stored in sterile 1.5 mL polypropylene tubes at −20 °C until molecular analyses.

To verify the presence of amplifiable DNA in the samples, internal control polymerase chain reaction (PCR) assays were performed targeting fragments of the mammalian cytochrome b (*CytB*) gene (Kocher et al., 1989) and the tick mitochondrial 16S rRNA gene (Mangold et al., 1998). DNA quality was assessed based on the successful amplification of these internal controls; therefore, no additional quantification methods were employed.

DNA from *Ehrlichia canis* strain São Paulo cultured in DH82 cells was used as the positive control in the PCR assays, while nuclease-free water was used as the negative control.

### Molecular screening and characterization for Anaplasmataceae agents

Positive samples in the abovementioned PCR assays were subjected to a PCR assay for Anaplasmataceae agents targeting a 360 bp fragment of the 16S rRNA gene with the primers *GE2’F2’* (5’-GTTAGTGGCAGACGGGTGAGT-3’) and *HE3* (5’-TATAGGTACCGTCATTATCTTCCCTAT-3’) (Almeida et al., 2013). For additional molecular characterization, samples that tested positive in the initial 16S rRNA screening were further analyzed using additional PCR assays targeting multiple genetic markers commonly employed in Anaplasmataceae phylogenetic studies: 16S rRNA (~1,400 bp) (Oh et al., 2009), 23S-5S rRNA ITS (~300 bp) (Rejmanek et al., 2012), *groEL* (~1,297 bp) (Liz et al., 2002), *groEL* (~680 bp), targeting different fragments of the gene, (Müller et al., 2018), and *gltA* (~561 bp) (Gofton et al., 2016).

PCR products were electrophoresed on 1.5% agarose gels prepared with 0.5× TBE buffer and stained with SYBR Safe^®^ (Invitrogen, Carlsbad, CA, USA). Electrophoresis was performed at 1-10 V/cm for 90 min. Gels were visualized using an LED transilluminator (Kasvi, São José dos Pinhais, PR, Brazil). Amplicons from positive samples with the highest intensity on agarose gels were purified for sequencing.

### Sequencing, BLASTn and phylogenetic analyses

Positive PCR products were purified using the Wizard^®^ SV Gel and PCR Clean-Up System (Promega, Madison, WI, USA). The concentration (ng/μL) of the DNA samples were assessed by optical spectrophotometry in a NanoDrop™ One Microvolume UV-Vis device (Thermo Fisher Scientific, Massachusetts, USA), only samples with more than 10 ng/μL were sequenced. Sequencing was carried out using the BigDye™ Terminator v3.1 Cycle Sequencing Kit (Applied Biosystems, Foster City, CA, USA) at the Aggeu Magalhães Institute, Oswaldo Cruz Foundation (FIOCRUZ-PE). Sequencing reactions were performed bidirectionally on an ABI 3500xL Genetic Analyzer (Applied Biosystems), using the same primers as in the PCR assays. Although Sanger sequencing is most effective for fragments up to approximately 800-1,000 bp, the near-complete 16S rRNA gene (~1,400 bp) was successfully sequenced using overlapping reads generated with internal primers, which were subsequently assembled to obtain the final consensus sequence (Clarridge, 2004).

Sequences obtained were first submitted to a screening test using Phred-Phrap software version 23 (Ewing et al., 1998) to evaluate the electropherogram quality and to obtain consensus sequences from the alignment of sense and antisense sequences. The Nucleotide BLAST program was used to analyze nucleotide sequences (BLASTn), aiming to browse and compare them with sequences previously deposited in GenBank. All sequences that showed appropriate quality standards and identity with Anaplasmataceae were deposited in GenBank (accession number PV916322.1; PV926528.1; PV927477.1) and in Brazilian National System for the Management of Genetic Heritage and Associated Traditional Knowledge (SISGEN) (accession number AEFEE08). The sequences were aligned with sequences published in GenBank using MAFFT software, version 7 (Katoh et al., 2019).

Phylogenetic inferences were performed with IQ-Tree web server (available online: http://iqtree.cibiv.univie.ac.at/), based on the Maximum Likelihood (ML) method (Trifinopoulos et al., 2016). Clade support was evaluated through 1,000 bootstrap replicates. The best model of evolution was selected by the same program (IQ-Tree web server), under the Bayesian Information Criterion (BIC). For the 16S rRNA gene alignment (1,229 bp), the selected evolutionary model was TPM2u+I+G. For the 23S-5S rRNA ITS alignment (345 bp), the best-fit model was TIM3e+G. The trees were examined in Treegraph 2.0.56-381 beta (Stöver & Müller, 2010).

## Results

### Tick identification

A total of 300 ticks were collected from 37 mammals belonging to nine host species, comprising eight tick species. Detailed taxonomic identification data have been previously reported by Bittencourt et al. (2025). The most prevalent species was *A. sculptum*, accounting for 65.3% (196/300) of all collected specimens, including 123 nymphs, 40 males, and 33 females. The remaining identified species were *A. dubitatum* (12.7%; 38/300), *A. nodosum* (8.3%; 25/300), *Amblyomma ovale* (1.3%; 4/300), *Amblyomma calcaratum* (1.3%; 4/300), *Amblyomma longirostre* (1.3%; 4/300), *Rhipicephalus microplus* (1.0%; 3/300), and *Dermacentor nitens* (0.7%; 2/300). In addition, 8.0% (24/300) of the specimens were identified as *Amblyomma* spp. larvae.

From the total collected ticks, 69 specimens were selected for molecular analyses. The remaining specimens were deposited in the “Coleção Nacional de Carrapatos do Cerrado” (CNCC) Marcelo Bahia Labruna, at the School of Veterinary and Animal Science, Federal University of Goiás (accession numbers 104-120, additional details can be found in Bittencourt et al., 2025), to ensure taxonomic verification and to support future studies.

### Blood smear analysis

All blood smears prepared from the 32 mammalian samples were examined under light microscopy and showed no detectable morphological structures consistent with the Anaplasmataceae family, indicating the absence of visible bacteremia at the time of sampling.

### Anaplasmataceae agents DNA in ticks

Among the 69 ticks tested, all presented positive results in the tick mitochondrial 16S rRNA gene. In the screening evaluation of the 16S rRNA gene of Anaplasmataceae, 2.9% (2/69) ticks presented positive results: a female of *A. sculptum* parasitizing an anteater (*M. tridactyla*) and a nymph of *A. sculptum* parasitizing a crab-eating fox (*C. thous*). Both mammal host blood samples presented negative results in the PCR.

### Molecular screening for Anaplasmataceae agents in wild

All blood and tissue samples from wild mammals tested were positive in the endogenous control *CytB*. For blood samples, 11.3% (11/97) were positive in the screening PCR for Anaplasmataceae agents targeting a fragment of the 16S rRNA, including six mammal species (one *C. thous*, one *C. brachyurus*, two *H. hydrochaeris*, one *M. tridactyla*, five *S. gouazoubira*, and one *T. terrestris*) (Table 1).

Regarding tissue samples, 13.6% (3/22) were positive in the screening PCR for Anaplasmataceae agents. Positive samples comprised liver and spleen tissues from a single *H. hydrochaeris* and a liver sample from one *C. brachyurus*. Notably, all animals with positive tissue results had negative blood samples in the same PCR assay.

### Molecular characterization, BLASTn and phylogenetic analyses

Regarding gel-purified DNA from Anaplasmataceae-positive samples, only two samples (one from *S. gouazoubira* and one from *H. hydrochaeris*) yielded DNA concentrations above 10 ng/μL, both derived from blood samples. Although amplicons were observed in other PCR-positive samples, low DNA yield after gel purification combined with suboptimal template quality resulted in unreadable or mixed chromatograms. Consequently, only these two samples were successfully sequenced and included in the molecular characterization (Table 2).

**Table 2:** BLASTn results of the *Anaplasma* spp. 16S rRNA and 23S-5S rRNA ITS sequences detected in wild mammals from the Midwestern region of Brazil.

**Sample no. (GenBank accession no.) wild mammal species**	**Molecular Marker**	**Size (bp)**	**Percent identity**	**Query cover (%)/E-value**	**Sequence best match location/host (GenBank accession no.)**
Sample 23 (PV926528.1; PV927477.1) *Subulo gouazoubira*	16S rRNA	1213	100%	100%/0.0	*Anaplasma* sp. South Africa/*Bos taurus* (OQ348132.1)
	23S-5S rRNA ITS	337	92.63%	100%/1X10^-131^	*Anaplasma platys* Saint Kitts and Nevis/*Canis lupus familiaris* (CP046391.1)
Sample 45 (PV916322.1) *Hydrochoerus hydrochaeris*	16S rRNA	250	100%	100%/0.0	*“Candidatus* Anaplasma boleense” Argentina / *Amblyomma tigrinum* (OR885906.1)

The sequence obtained from capybara (*H. hydrochaeris*) showed 100% identity with “*Ca.* Anaplasma boleense” (GenBank accession number OR885906.1), previously detected in *Amblyomma tigrinum* from *Lycalopex gymnocercus* in Argentina. The obtained sequence (250 bp in length) was deposited in GenBank under the accession number PV916322.1 (Table 2). However, this sample tested negative in all additional PCR assays targeting the near- complete 16S rRNA gene, 23S-5S rRNA ITS, *groEL*, and *gltA* genes, precluding further molecular characterization of the strain.

A large fragment of the 16S rRNA of *Anaplasma* spp. was amplified and successfully sequenced (1,213 bp) from the gray brocket deer blood and deposited in GenBank under the accession number PV926528.1. BLASTn analysis revealed 100% identity with *Anaplasma* sp. (GenBank accession number OQ348132.1) previously detected in blood samples from a mixed-breed *Bos taurus* from South Africa. This sample was also positive for the 23S-5S rRNA intergenic spacer (ITS), generating a 337 bp sequence that was deposited in GenBank under accession number PV927477.1, BLAST analysis revealed 92.6% identity with *Anaplasma platys* (GenBank accession number CP046391.1), previously reported in dogs from Saint Kitts and Nevis, representing the closest match. However, this sample tested negative in additional PCR assays targeting the *groEL* and *gltA* genes, preventing further molecular characterization.

**Figure 1:**
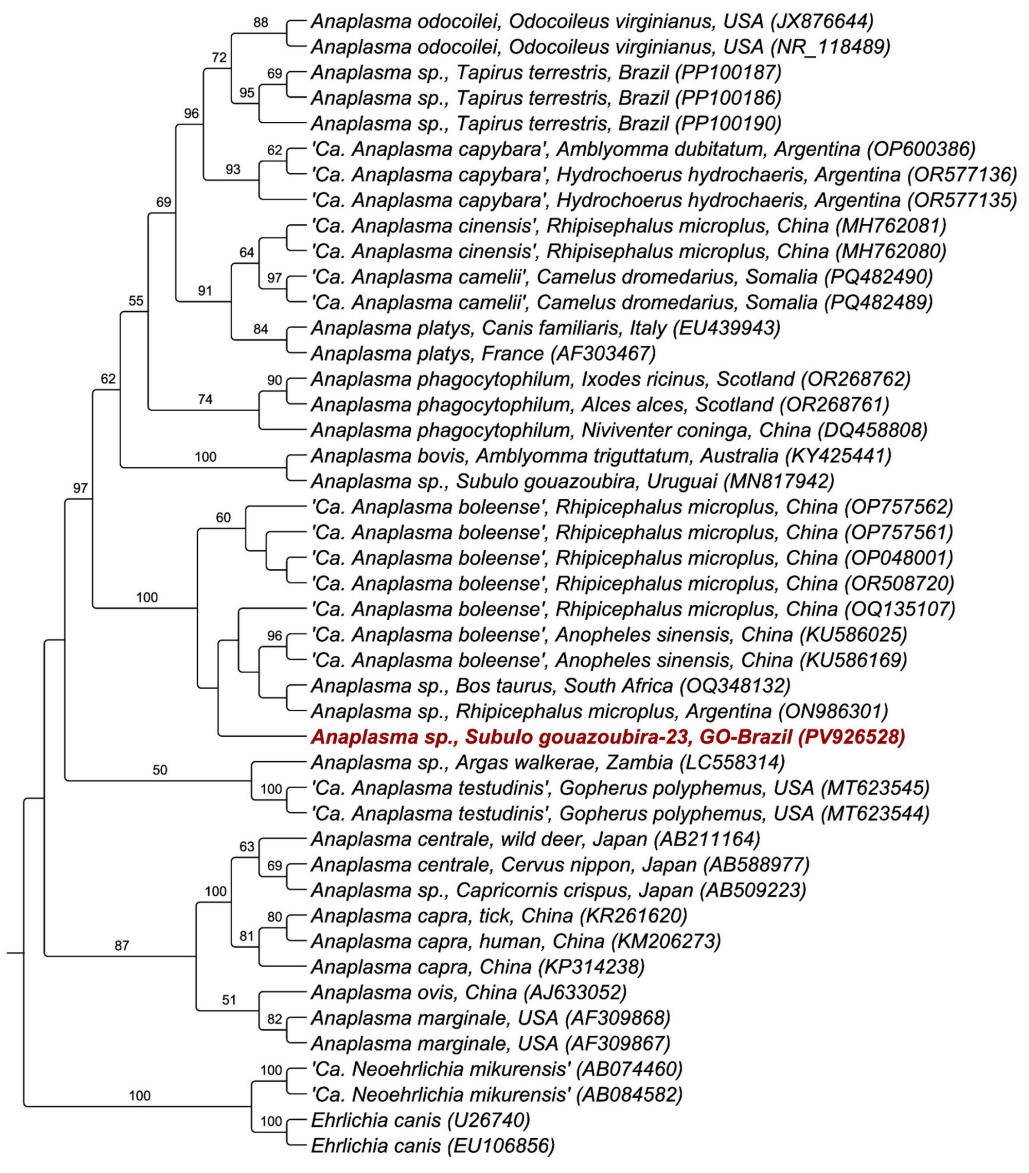
Maximum likelihood phylogenetic analysis based on the 16S rRNA gene (1,229 bp). Sequence obtained in the present study were highlighted in red. The tree was constructed using the TPM2u+I+G evolutionary model. Bootstrap values (>50) are shown at the nodes. ‘*Candidatus* Neoehrlichia mikurensis’ and *Ehrlichia canis* were used as an outgroup to root the tree.

**Figure 2:**
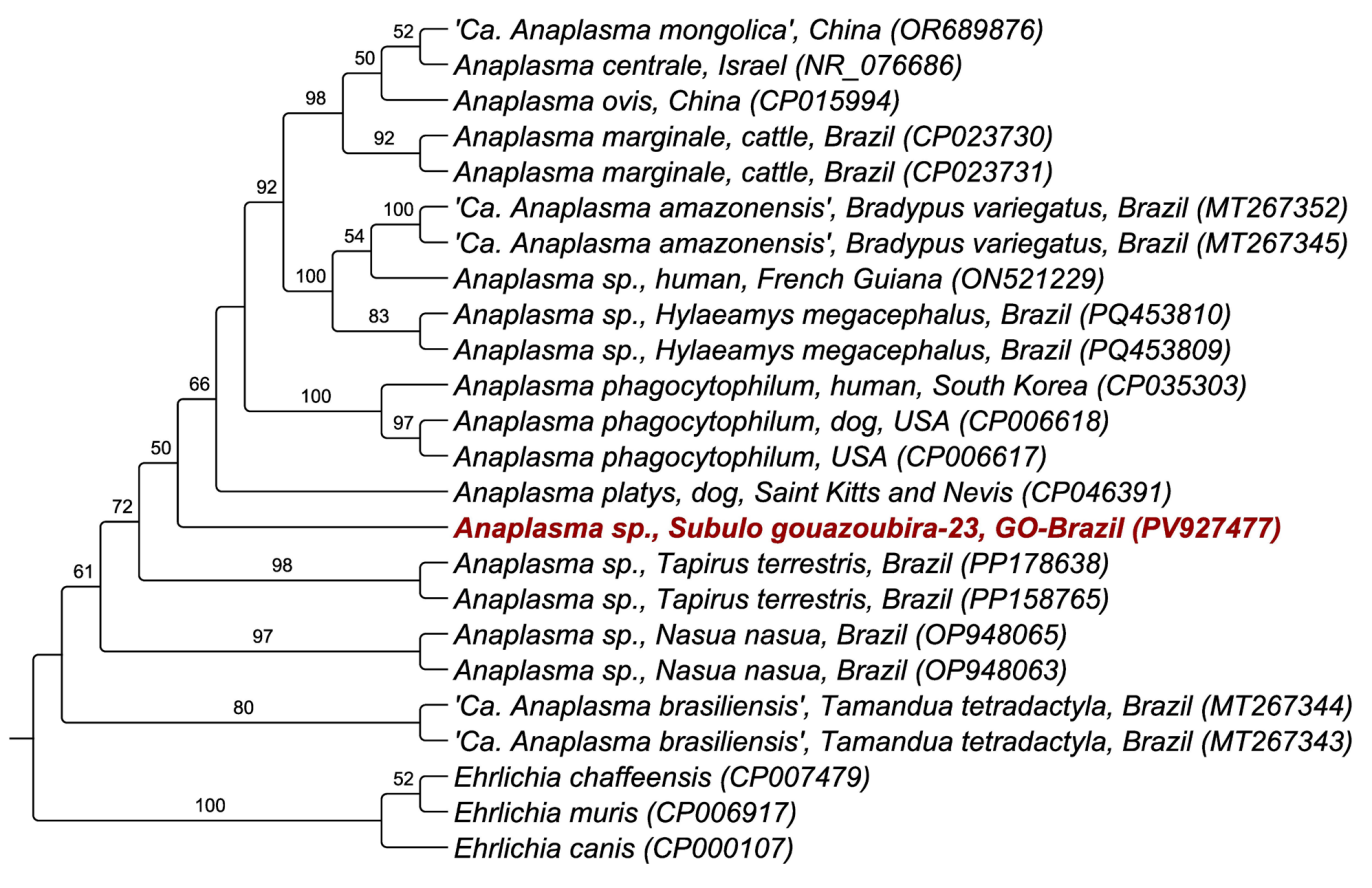
Maximum likelihood phylogenetic analysis based on the internal transcribed spacer *23S-5S* rRNA ITS region (345bp). Sequence obtained in the present study was highlighted in red. The tree was constructed using the TIM3e+G evolutionary model. Bootstrap values (>50) are shown at the nodes. *Ehrlichia* spp. were used as an outgroup to root the tree.

Maximum Likelihood phylogenetic analysis based on the near-complete 16S rRNA gene was performed to investigate the evolutionary relationships of the sequences obtained in this study with those of other *Anaplasma* species worldwide (Figure 1). The sequence obtained from the gray brocket deer clustered within the “*Ca.* A. boleense” clade, alongside sequences previously reported from ticks (*Rhipicephalus microplus*) and mosquitoes (*Anopheles sinensis*) from China. This clade was well-supported by a bootstrap value of 100%, confirming the phylogenetic positioning of the detected genotype within the “*Ca.* A. boleense” group. Additionally, a ML phylogenetic tree based on the ITS 23S-5S rRNA region was constructed to analyze the *Anaplasma* sequence obtained from *S. gouazoubira* (Figure 2). The topology of the tree also showed that this sequence is phylogenetically related to other *Anaplasma* spp. recently detected in wild mammals in Brazil, including *Anaplasma* spp. from *T. terrestris* and *N. nasua*, as well as “*Ca.* Anaplasma brasiliensis” detected in *T. tetradactyla.* But the sequence formed a distinct and well-supported clade, separated from all other *Anaplasma* species included in the analysis, including *A. platys* and *A. phagocytophilum*. No 23S-5S rRNA ITS sequences from “*Ca.* A. boleense” were available in public databases for comparison.

## Discussion

This study reports the occurrence of Anaplasmataceae agents in blood, tissue, and ticks from wild mammals in midwestern region of Brazil. The presence of these bacteria has been previously reported in Brazil, and it is well established that wild mammals play important roles in maintaining and spreading these bacteria in natural environments (Sousa et al., 2017; Braga et al., 2018; Calchi et al., 2020; Santana et al., 2022). The genotypes detected may represent host-adapted lineages or strains closely related to those previously reported in domestic animals (Guillemi et al., 2016). This is particularly relevant in Goiás, midwestern Brazil, the state is predominantly covered by the Cerrado biome, the second largest biome in South America, a recognized biodiversity hotspot, where anthropogenic pressures increase the interface between wildlife, livestock, and humans (Mongruel et al., 2017; Ramos et al., 2020; Silva et al., 2024; Silva & Lacher, 2020).

In this study, 11.3% (11/97) of mammals tested positive in the 16S rRNA screening PCR for Anaplasmataceae. It is essential to consider that captive animals are more likely to receive veterinary care and are generally less exposed to ticks, which may result in lower infection rates compared to free-ranging populations (Bastos et al., 2016; Bittencourt et al., 2025). Also, the PCR detection of *Anaplasma* DNA in tissue samples (liver and spleen), while blood samples were negative, as observed in a capybara and a maned wolf, this can be explained by low‑level bacteremia limit of molecular assays, yet the pathogen can persist and concentrate in reticuloendothelial organs such as the liver and spleen, these findings were observed in other studies with wild animals, where spleen and liver samples yielded higher *Anaplasma* prevalence than blood (Karbowiak et al., 2015; Myczka et al., 2021). This finding further indicates that tissue samples represent a valuable complementary source to blood samples for the surveillance and estimation of the prevalence of Anaplasmataceae agents in wildlife populations.

Among the positive wild animals, gray brocket deer stood out for its high positivity rate (41.6%), indicating a significant level of exposure to Anaplasmataceae agents. Some deer species are recognized as reservoirs for *A. phagocytophilum*, with the white-tailed deer (*Odocoileus virginianus*) serving as the primary reservoir in North America (Stuen et al., 2013). In Brazil, natural infections by *Anaplasma* species have been previously reported in deer, including *Anaplasma marginale* (Machado et al., 2006; Silveira et al., 2012, 2013, 2016) and genotypes closely related to *A. phagocytophilum* (Silveira et al., 2012; Mongruel et al., 2017)*, A. bovis* (Silveira et al., 2012; Mongruel et al., 2017), *A. platys* (Sacchi et al., 2012; Silveira et al., 2012), and *E. chaffeensis*, (Machado et al., 2006). Notably, phylogenetic analyses of *Anaplasma* spp. in brocket deer sampled in southern Brazil have revealed considerable genetic diversity and topological incongruences among gene trees, suggesting the presence of distinct species within this host group (Mongruel et al., 2017). While 16S rRNA sequences detected in *S. gouazoubira* clustered with *A. bovis, groESL* sequences grouped with genotypes detected in wild deer from Japan (Mongruel et al., 2017). On the other hand, *A. marginale* and *Anaplasma* spp. related to *A. platys/A. phagocytophilum* were detected in brocket deer sampled in southeastern Brazil (Silveira et al., 2012). This genetic diversity reinforces *S. gouazoubira* as an important host for undescribed *Anaplasma* species adapted to the Neotropical fauna.

The gray brocket deer (*S. gouazoubira*) is the most abundant and widely distributed deer species in the Neotropics (Bernegossi et al., 2023). Its adaptability, combined with its proximity to livestock and human-modified environments, suggests a potential role in the local maintenance and spreading of *Anaplasma* spp.

The near-complete 16S rRNA sequence obtained from *S. gouazoubira* clustered within a well-supported clade alongside *Anaplasma* spp. previously detected in *R. microplus* ticks from Argentina and cattle from South Africa (Figure 1). Although closely related to other *Anaplasma* sequences identified in domestic and wild hosts across South America, the sequence from gray brocket deer, formed a distinct branch within this clade, suggesting a potentially novel or regionally adapted lineage circulating in Neotropical cervids. Phylogenetic analysis based on the ITS 23S-5S rRNA region further supported this interpretation. The sequence obtained in this study formed a separate, well-supported clade, clearly distinct from *A. platys*, *A. phagocytophilum*, and other reference sequences (Figure 2). These results indicate the presence of an uncharacterized *Anaplasma* lineage in *S. gouazoubira*, which is phylogenetically distinct from currently described species. Although it shows some similarity to *‘Ca.* A. boleense’, a definitive taxonomic assignment remains challenging because of the limited availability of ITS 23S-5S rRNA reference sequences for this taxon in public databases.

Two out of four capybaras sampled were positive for Anaplasmataceae, reinforcing their potential epidemiological relevance as a host for tick-borne pathogens. They are widely recognized for their adaptability to urban and peri-urban environments and have already been implicated as a key amplifier host for *Rickettsia rickettsii*, the etiologic agent of Brazilian spotted fever, through their association with *A. sculptum* ticks (Luz et al., 2019). However, studies investigating their role in the epidemiology of Anaplasmataceae agents are still scarce.

In this study, a short sequence of the 16S rRNA showing high identity to ‘*Ca.* A. boleense’ was identified in *H. hydrochaeris*. *‘Ca.* A. boleense’ is a recently described *Anaplasma* genotype initially reported in ticks and vertebrate hosts in Asia (Khan et al., 2024). Although this finding may suggest a potential expansion of the known host range for this genotype, we acknowledge that the short fragment size (250bp) and the inability to amplify additional genetic markers limit the taxonomic and phylogenetic resolution of this result. Therefore, this finding should be interpreted as preliminary detection of an agent related to ‘*Ca.* A. boleense’ in capybaras, rather than as a definitive species identification. This limitation may be due to low bacterial loads, degraded DNA, or substantial genetic divergence in primer-binding regions, as previously reported by Vieira et al. (2022). Furthermore, the short 16S rRNA fragment obtained (250 bp) restricts the depth of phylogenetic inference. Also, previous findings that reported the presence of a *Anaplasma* species in capybaras, with 16S rRNA sequences showing up to 97.9% identity with *A. phagocytophilum* and phylogenetic proximity to *Anaplasma odocoilei* (Vieira et al., 2022). Additionally, phylogenetic analyses based on the 16S rRNA gene in goats from Maranhão, Brazil, also revealed *Anaplasma* genotypes clustering with ‘*Ca.* A. boleense’, *A. platys*, and *A. marginale*, supporting the findings of the widespread and host-diverse circulation of *Anaplasma* spp. in South America (Silva et al., 2024).

Other positive hosts included *C. thous* and *C. brachyurus*, both wild carnivores already recognized for their role in maintaining and disseminating ticks and associated tick-borne pathogens (Labruna et al., 2005; André et al., 2012; Sousa et al., 2017; Ramos et al., 2020). These species are frequently exposed to a diversity of tick species and hemoparasites, serving as bridge hosts between wild environments and areas with human and domestic animal presence (André, 2018). Similarly, *T. terrestris* and *M. tridactyla* were also found to be positive. Both species are known to exhibit high tick burdens and are susceptible to multiple tick-borne agents, including a putative novel *Anaplasma* genotype in lowland tapirs (Mongruel et al., 2022, 2024) and *Ehrlichia* genotypes closely related to canine-associated *E. canis* (Calchi et al., 2020) and cattle-associated *E. minasensis* (Sada et al., 2024) in giant anteaters, reinforcing their potential role in the epidemiology of hemoparasites in the Cerrado biome (Bittencourt et al., 2025; Guillemi et al., 2016; Mongruel et al., 2024).

A limitation of this study is that molecular analyses were primarily focused on the most abundant tick species, *A. sculptum*. Therefore, the conclusions mainly reflect pathogen circulation within the dominant tick species in the study area and may underestimate the presence of tick-borne agents circulating in less abundant or rarer tick species. Although the overall positivity rate in ticks was low (2.9%), this confirms the circulation of Anaplasmataceae within the local tick population and underscores their possible role as vectors. However, this finding should be interpreted with caution, as the detection of pathogen DNA in whole ticks does not necessarily indicate that these ticks are competent vectors (Gerardi et al., 2019). Given that ticks were removed from their hosts during blood feeding, the detected DNA may reflect the presence of infected host blood within the tick gut rather than an established infection in the tick. The low detection rate may be attributed to the captive management of the animals, which reduces natural exposure to infected ticks.

Additionally, co-infections with Anaplasmataceae and piroplasmids were detected in 5.1% (5/97) of the animals using the same blood samples analyzed in the present study, which had been previously examined for piroplasmids by Bittencourt et al. (2025). These co-infections occurred in three *S. gouazoubira*, one *H. hydrochaeris*, and one *T. terrestris*. These findings corroborate observations by Perles et al. (2023), who reported high rates of co-infection involving hemoparasites in wild mammals from Brazil. Co-infections are increasingly recognized as common in wildlife, reflecting the complex network of vector-host-pathogen interactions in natural ecosystems. Notably, the same capybara sample that yielded the *Anaplasma* sequence also showed identity with *Babesia goianiaensis* (Bittencourt et al., 2025), reinforcing the ecological significance of this mammal as a host for multiple tick-borne pathogens.

Despite the increasing number of reports describing tick-borne pathogens in wildlife based on molecular detection, the ecological role of these hosts as reservoirs or maintenance hosts remains poorly understood. Most available data are based solely on pathogen DNA detection, whereas information on pathogen morphology, pathogenesis, clinical relevance, and epidemiological dynamics remains scarce. Furthermore, the vectors of many of these agents have not yet been identified, underscoring the need for integrative approaches that combine molecular, ecological, clinical, and vector competence data to better elucidate host-pathogen-tick interactions, as well as their potential health and conservation implications for wildlife.

This study enhances our understanding of hemoparasites in wild mammals within midwestern, Brazil. Our findings underscore the importance of continued surveillance of emerging tick-borne agents, with direct implications for wildlife conservation, ecosystem health, and the prevention of zoonotic diseases. This is particularly relevant in the context of the Cerrado, a biome characterized by intense habitat fragmentation and increasing interfaces between wildlife, domestic animals, and humans (da Silva & Lacher, 2020; Ramos et al., 2020).

## Conclusion

This study expands the current knowledge on the diversity and circulation of Anaplasmataceae bacteria in wild mammals from Goiás state, midwestern Brazil. The detection of these bacteria in multiple species, particularly in *S. gouazoubira* and *H. hydrochaeris*, suggests a potential role of these wild animals in the maintenance of tick-borne pathogens. Phylogenetic analyses revealed genetically diverse genotypes and possibly undescribed *Anaplasma* lineages, specifically a 16S rRNA genotype and 23S-5S rRNA ITS lineage in *S. gouazoubira.* Additionally, molecular analysis identified a 16S rRNA genotype related to ‘*Ca.* A. boleense’ in *H. hydrochaeris*. These findings underscore the complexity of *Anaplasma* genotypes that circulates in the Cerrado biome, highlighting the importance of including wild mammals in surveillance programs and reinforcing the need for further studies to understand the eco-epidemiology, genetic diversity, and one health implications of Anaplasmataceae in South American ecosystems.

## Data Availability

The data supporting the findings of this study are available within the article and may be provided upon formal request to the corresponding author. As well as all material is in their respective collections and are available for public consultation.
